# Factors associated to potentially inappropriate prescribing in older patients according to STOPP/START criteria: MoPIM multicentre cohort study

**DOI:** 10.1186/s12877-021-02715-8

**Published:** 2022-01-11

**Authors:** Marisa Baré, Marina Lleal, Sara Ortonobes, Maria Queralt Gorgas, Daniel Sevilla-Sánchez, Nuria Carballo, Elisabet De Jaime, Susana Herranz, on behalf of the MoPIM study group

**Affiliations:** 1 Institutional Committee for the Improvement of Clinical Practice Adequacy, Consorci Corporació Sanitària Parc Taulí, Parc Taulí 1, 08208 Sabadell, Catalonia Spain; 2 Health Services Research on Chronic Patients Network (REDISSEC), Sabadell, Spain; 3 Pharmacy Department, Consorci Corporació Sanitària Parc Taulí, Sabadell, Spain; 4 Pharmacy Department, Consorci Hospitalari de Vic, Vic, Spain; 5 Pharmacy Department, Consorci Parc de Salut MAR, Barcelona, Spain; 6 Geriatrics Department, Consorci Parc de Salut MAR, Barcelona, Spain; 7 Acute Geriatric Unit, Consorci Corporació Sanitària Parc Taulí, Sabadell, Spain

**Keywords:** Potentially inappropriate prescribing, STOPP/START version 2, polypharmacy, older patient

## Abstract

**Objectives:**

The objectives of the present analyses are to estimate the frequency of potentially inappropriate prescribing (PIP) at admission according to STOPP/START criteria version 2 in older patients hospitalised due to chronic disease exacerbation as well as to identify risk factors associated to the most frequent active principles as potentially inappropriate medications (PIMs).

**Methods:**

A multicentre, prospective cohort study including older patients (≥65) hospitalized due to chronic disease exacerbation at the internal medicine or geriatric services of 5 hospitals in Spain between September 2016 and December 2018 was conducted. Demographic and clinical data was collected, and a medication review process using STOPP/START criteria version 2 was performed, considering both PIMs and potential prescribing omissions (PPOs). Primary outcome was defined as the presence of any most frequent principles as PIMs, and secondary outcomes were the frequency of any PIM and PPO. Descriptive and bivariate analyses were conducted on all outcomes and multilevel logistic regression analysis, stratified by participating centre, was performed on the primary outcome.

**Results:**

A total of 740 patients were included (mean age 84.1, 53.2% females), 93.8% of them presenting polypharmacy, with a median of 10 chronic prescriptions. Among all, 603 (81.5%) patients presented at least one PIP, 542 (73.2%) any PIM and 263 (35.5%) any PPO. *Drugs prescribed without an evidence-based clinical indication* were the most frequent PIM (33.8% of patients); *vitamin D supplement in older people who are housebound or experiencing falls or with osteopenia* was the most frequent PPO (10.3%). The most frequent active principles as PIMs were proton pump inhibitors (PPIs) and benzodiazepines (BZDs), present in 345 (46.6%) patients. This outcome was found significantly associated with age, polypharmacy and essential tremor in an explanatory model with 71% AUC.

**Conclusions:**

PIMs at admission are highly prevalent in these patients, especially those involving PPIs or BZDs, which affected almost half of the patients. Therefore, these drugs may be considered as the starting point for medication review and deprescription.

**Trial registration number:**

NCT02830425

**Supplementary Information:**

The online version contains supplementary material available at 10.1186/s12877-021-02715-8.

## Background

Older patients with multiple morbidities and medication requirements pose a challenge to the prescribing physicians. In addition to possible drug-drug or drug-disease interactions, these patients present age-related physiological changes in drug pharmacokinetics and pharmacodynamics, as well as other factors that can influence prescription such as cognitive impairment, functional difficulties or geriatric syndromes [1, 2].

Considering this, the term potentially inappropriate prescribing (PIP) is being widely used to describe a range of situations in which prescribing should be revised, particularly in geriatric patients. PIP includes potentially inappropriate medication (PIM) which, together with polypharmacy, are well-known risk factors for adverse drug events [3, 4], and also includes potential prescribing omissions (PPO), which increase the probability of not taking essential medication [5, 6].

There are several tools to identify and evaluate PIP [7]. Among all, the explicit criteria STOPP/START (Screening Tool of Older Person's potentially inappropriate Prescriptions / Screening Tool to Alert doctors to Right Treatment) [8], which includes PIMs and PPOs, were the first European criteria and are currently the most used and validated in European elderly people [9]. After the 1^st^ version, containing 84 criteria, a 2^nd^ version with 114 criteria was later developed, expanding the explicit criteria as well as incorporating three implicit criteria [10].

In recent years, many studies have been published using these criteria to assess prescription adequacy in different settings, such as primary care, socio-health centres, nursing homes and hospitals [7, 11–14]. Additionally, several studies have identified factors associated with the number or presence of PIM or PPO, such as polypharmacy, number of morbidities or age, as well as associated PIM or PPO to clinical outcomes such as hospitalization or mortality [15–17].

However, to the best of our knowledge, there are currently no studies evaluating PIP and its associated factors in a cohort of older patients admitted to hospital due to chronic condition exacerbation. This constitutes an especially vulnerable and complex group of patients that come from the community but end up hospitalized, and may present avoidable, inappropriate prescriptions at admission. Moreover, despite the high prevalence of multimorbidity in older patients, there are no studies evaluating a comprehensive list of chronic conditions as possible risk factors for PIP nor any studies focusing on the most frequent active principles as PIMs, which would be really helpful to develop more efficient strategies.

Thus, the objectives of the present analyses are to estimate the frequency of PIMs and PPOs at admission according to STOPP/START criteria (2^nd^ version) and to identify risk factors associated to the most frequent active principles as PIMs, evaluating sociodemographic, clinical and pharmacological variables in older patients admitted to hospital because of an exacerbation of their chronic conditions. These analyses are part of a larger study, named MoPIM (Morbidity, Potentially Inappropriate Medication), with various objectives related to multimorbidity, PIP and adverse drug reactions in these patients.

## Methods

### Design and setting

A multicentre, prospective cohort study including older patients hospitalized at the internal medicine or geriatric services at five general teaching hospitals in three different regions of Spain between September 2016 and December 2018 was conducted. The detailed protocol was previously published [18].

For the purposes of this study, older patients (≥65 years old) admitted because of an exacerbation of their chronic pathology were included. Patients referred to home hospitalization, admitted because of an acute process, or with a fatal outcome expected at admission were not included.

No written informed consent was deemed necessary for this study, according to the independent ethics committee.

### Data acquisition and variables

The following sociodemographic and clinical data was retrieved by the clinical team responsible for the patient: patient's code, centre, date of birth, sex, functional status just before entering the hospital (Barthel Index) [19], household (alone, with relatives or other people, in a nursing home) and existence of any contact with healthcare services (primary care, emergencies, hospital admission, outpatient care, home care) in the 3 months prior to hospitalization due to exacerbation of any chronic disease. Chronic active conditions were recorded from a consensual list of 64 conditions, which included risk factors and all chronic diseases of the Charlson Comorbidity Index [20].

Regarding pharmacological variables, the number of chronic medications in the electronic prescription at the time of admission and the STOPP/START criteria detected upon admission, with the active principle involved, were collected by the pharmacist of the team. This medication review process is routinely conducted in all participating centres. Medication was only considered chronic if prescribed at least 3 months before admission, and creams, ointments, healing material and over-the-counter medicines were not considered. Active principles were considered individually when registering STOPP/START criteria, regardless of the administered drug combinations.

### Sampling and analysis

The estimated sample of 800 patients (see protocol [18]) could not be reached due to organizational reasons in one of the participating centres. Patients included were proportionally distributed to the annual volume of hospitalizations at the internal medicine and/or geriatric services of each centre.

For the purposes of the analyses, age was categorized as 65-74, 75-89, or >89 years and the number of chronic conditions was categorized as 1-7, 8-13 or 14-22. These categorizations were established by using the catpredi() R function [21], which provides the optimal cut-off points for categorization of quantitative variables based on the relationship between these variables and the outcome (presence of any of the most frequent active principles as PIMs). The Updated Charlson Comorbidity Index [22] was calculated, adjusted by age and categorized by tertiles (2-6, 7-8 and 9-14). Barthel Index was categorized as independency (100 points), minimal dependency (60-95), moderate dependency (40-55), severe dependency (20-35) and complete dependency (<20) [23].

Some chronic conditions were grouped according to clinical criteria, as in Baré et.al. [24] Eventually, 50 chronic conditions were analysed.

Polypharmacy was defined as the chronic consumption of five or more drugs [25]. On top of that, another categorisation was defined at 10 drugs and patients were therefore classified as presenting ‘oligopharmacy’ (<5 drugs), ‘moderate polypharmacy’ (5-9 drugs), and ‘excessive polypharmacy’ (≥10 drugs).

All STOPP/START criteria were assessed, except for START criteria I (vaccines), due to difficulties of some centres in accessing the information (not registered in the electronic prescription). Regarding the implicit criterion STOPP A1 and given its high frequency, it was divided into the following categories according to the active principle involved: proton pump inhibitors (PPIs), hypolipidemics, analgesics, aspirin, antihypertensives and others.

Descriptive analyses were performed for all variables. Bivariate analyses were conducted to assess possible associations between sociodemographic/clinical variables and PIP related outcomes (any PIM, any PPO, any most frequent active principles as PIMs) by the chi-square test.

Multilevel logistic regression analysis was performed on the primary outcome (presence of any most frequent active principles as PIMs). Hospital centre was set as a level (random effect) in order to account the possibility that in each hospital location, the prescriptive practices of all professionals in each area may be different and lead to some variability in PIP. Explanatory variables (fixed effect) were chosen if p<0.05 in the bivariate analysis. The final model was determined by a stepwise algorithm, with a minimal Akaike Information Criteria value, and its Area Under the Curve (AUC) was calculated.

All analyses were performed with R (R Foundation for Statistical Computing, Vienna, v3.6.0).

## Results

### Description of sociodemographic and clinical data

A consecutive sample of 740 patients aged ≥65 years was obtained, with a mean age of 84.1 years (SD±7.0) and a 53.2% of females. Sociodemographic and clinical variables are summarised in Table 1. The median number of chronic conditions was 8 (interquartile range (IQR) 6-11), ranging from 1 to 22, and the number of chronic prescriptions ranged from 0 to 28, with a median of 10 (IQR 7-13). Most (93.8%) patients presented polypharmacy; precisely, 259 (35%) patients had moderate polypharmacy, and 435 (58.8%) displayed excessive polypharmacy.

**Table 1:** Descriptive and bivariate (chi-square test) statistics of sociodemographic and clinical data related to the presence of any PIP, PIM, PPO and most frequent active principles as PIMs (proton pump inhibitors or benzodiazepines), according to STOPP/START criteria. N, % and 95% Confidence Intervals (95% CI) are shown, as well as chi-square p-value

Sociodemographic and clinical variables		Total	Any STOPP PIM			Any START PPO			Any most frequent active principles as PIMs (PPI/ BZD)		
		N (%)	N (%)	95% CI	p-value	N (%)	95% CI	p-value	N (%)	95% CI	p-value
Total		740 (100)	542 (73.2)	69.9-76.3	-	263 (35.5)	32.2-39.1	-	345 (46.6)	43.1-50.2	-
Age	65-74	81 (10.9)	52 (64.2)	53.3-73.8	0.072	13 (16.1)	9.6-25.5	**0.001**	26 (32.1)	22.9-42.9	**0.018**
	75-89	495 (66.9)	374 (75.6)	71.6-79.1		186 (37.6)	33.4-41.9		243 (49.1)	44.7-53.5	
	>89	164 (22.2)	116 (70.7)	63.4-77.2		64 (39.0)	31.9-46.7		76 (46.3)	38.9-54	
Sex	Female	394 (53.2)	303 (77.0)	72.5-80.8	**0.016**	142 (36)	31.5-40.9	0.762	194 (49.2)	44.3-54.2	0.128
	Male	346 (46.8)	239 (69.1)	64-73.7		121 (35)	30.1-40.1		151 (43.6)	38.5-48.9	
Barthel Index	< 20	90 (12.2)	64 (71.1)	61-79.5	**0.001**	24 (26.7)	18.6-36.6	**0.007**	39 (43.3)	33.6-53.6	**< 0.001**
	20-35	76 (10.3)	64 (84.2)	74.4-90.7		31 (40.8)	30.4-52		42 (55.3)	44.1-65.9	
	40-55	124 (16.8)	93 (75.0)	66.7-81.8		43 (34.7)	26.9-43.4		63 (50.8)	42.1-59.4	
	60-95	294 (39.7)	225 (76.5)	71.4-81		123 (41.8)	36.3-47.5		152 (51.7)	46-57.4	
	100	156 (21.1)	96 (61.5)	53.7-68.8		42 (26.9)	20.6-34.4		49 (31.4)	24.6-39.1	
uCCI	2-6	280 (37.8)	202 (72.1)	66.6-77.1	0.511	86 (30.7)	25.6-36.3	**0.007**	128 (45.7)	40-51.6	0.546
	7-8	279 (37.7)	211 (75.6)	70.3-80.3		119 (42.6)	37-48.5		80 (44.2)	37.2-51.5	
	9-14	181 (24.5)	129 (71.3)	64.3-77.4		58 (32)	25.7-39.2		137 (49.1)	43.3-54.9	
Household	With relatives / other people	523 (70.7)	381 (72.9)	68.9-76.5	0.689	183 (35)	31-39.2	0.459	60 (49.2)	40.5-57.9	0.532
	Nursing home	95 (12.8)	73 (76.8)	67.4-84.2		31 (32.6)	24-42.6		48 (50.5)	40.6-60.4	
	Alone	122 (16.5)	88 (72.1)	63.6-79.3		49 (40.2)	31.9-49		237 (45.3)	41.1-49.6	
Prior exacerbation	No	225 (30.4)	163 (72.4)	66.3-77.9	0.746	80 (35.6)	29.6-42	0.995	99 (44)	37.7-50.5	0.345
	Yes (total)	515 (69.6)	379 (73.6)	69.6-77.2		183 (35.5)	31.5-39.8		246 (47.8)	43.5-52.1	
	Primary care	342 (46.2)	251 (73.4)	68.5-77.8	0.933	128 (37.4)	32.5-42.7	0.32	170 (49.7)	44.4-55	0.119
	Emergencies	263 (35.5)	199 (75.7)	70.1-80.5	0.269	95 (36.1)	30.6-42.1	0.806	136 (51.7)	45.7-57.7	**0.039**
	Hospital admission	193 (26.1)	144 (74.6)	68-80.2	0.618	68 (35.2)	28.8-42.2	0.917	93 (48.2)	41.2-55.2	0.612
	Outpatient care	8 (1.1)	5 (62.5)	30.6-86.3	0.49	0 (0)	0-32.4	**0.035**	2 (25)	7.1-59.1	0.218
	Home hospitalization	14 (1.9)	12 (85.7)	60.1-96	0.287	4 (28.6)	11.7-54.6	0.582	9 (64.3)	38.8-83.7	0.181
Polymedication	Oligopharmacy (0-4)	46 (6.2)	16 (34.8)	22.7-49.2	**< 0.001**	19 (41.3)	28.3-55.7	0.367	10 (21.7)	12.3-35.6	**< 0.001**
	Moderate polypharmacy (5-9)	259 (35.0)	177 (68.3)	62.4-73.7		98 (37.8)	32.1-43.9		106 (40.9)	35.1-47	
	Excessive polypharmacy (10+)	435 (58.8)	349 (80.2)	76.2-83.7		146 (33.6)	29.3-38.1		229 (52.6)	47.9-57.3	
N° chronic conditions	1-7	303 (41.0)	200 (66.0)	60.5-71.1	**< 0.001**	102 (33.7)	28.6-39.2	0.657	124 (40.9)	35.5-46.5	**< 0.001**
	8-13	374 (50.5)	288 (77.0)	72.5-81		137 (36.6)	31.9-41.6		45 (71.4)	59.3-81.1	
	14-22	63 (8.5)	54 (85.7)	75-92.3		24 (38.1)	27.1-50.4		176 (47.1)	42.1-52.1	

PIP: potentially inappropriate prescribing. PIM: potentially inappropriate medication. PPO: potential prescribing omission. PPI: proton pump inhibitor. BZD: benzodiazepine. uCCI: updated Charlson Comorbidity Index.

### Potentially inappropriate prescribing

At least one PIP was reported in 603 (81.5%, 95% confidence interval (CI) 78.5-84.1) patients. The number of PIPs ranged from 0 to 8, with a median of 2 (IQR 1-3).

Regarding PIMs, 542 (73.2%, 95% CI 69.9-76.3) patients presented at least one. The median number of PIMs was 1 (IQR 0-2), ranging from 0 to 8, and 216 (29.2%) patients had one PIM, 148 (20%) had two PIMs, 87 (11.8%) had three PIMs, and 91 (12.3%) had four or more PIMs.

**Fig. 1:**
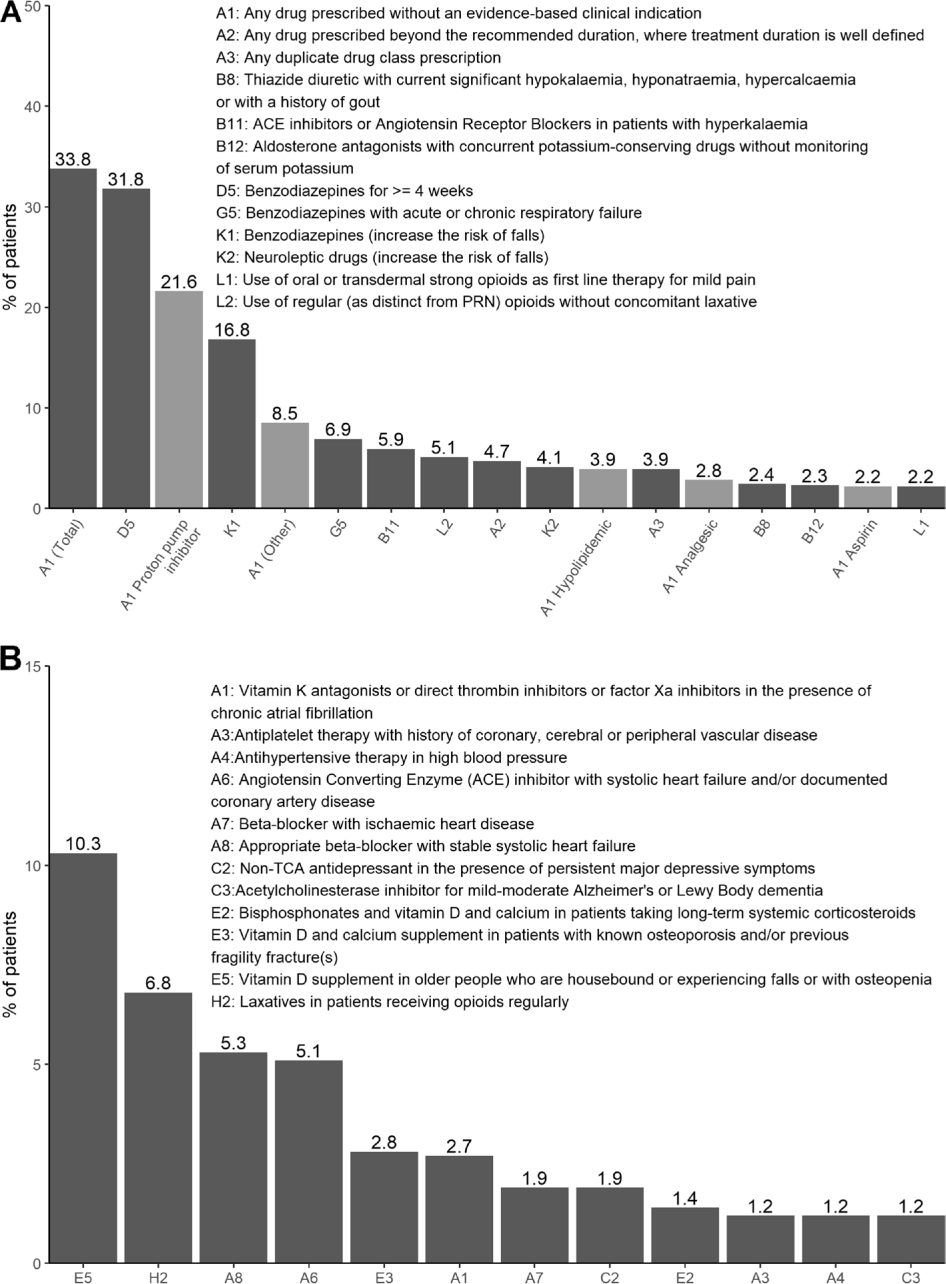
% of patients presenting the following STOPP/START criteria. A: Potentially inappropriate medications (PIMs) found in most patients according to STOPP criteria (present in >2% of the patients). Subcategories of criterion A1 are shown in mild grey. B: Potential prescribing omissions (PPOs) found in most patients according to START criteria (present in >1% of the patients).

*Drugs prescribed without an evidence-based clinical indication* were the most frequent PIM (STOPP criterion A1, in 33.8% of patients, many of them having multiple PIMs in this criterion, and accounting for 25.7% of the total number of PIMs). Detailed information of the active principles registered within this criterion can be found in Supp. Table. 1. Most frequent PIMs are represented in Figure 1A, relative to the total of patients, and all PIMs detected are shown in Supp. Table 2, relative to the total number of PIMs. Regarding the type of active principle involved, PIMs related to PPIs (STOPP criteria A1 or F2) were present in 22.6% of the patients.

**Table 2:** Bivariate analysis (chi-square test) between any PIM, any PPO, any most frequent active principles as PIMs (PPI/BZD) according to STOPP/START criteria and sociodemographic/clinical variables

Variable		Any STOPP PIM			Any START PPO			Any most frequent active principles as PIMs (PPI/ BZD)		
		N	%	p-value	N	%	p-value	N	%	p-value
Amputation	No	530	73.2	0.873	257	35.5	0.869	337	46.5	0.784
	Yes	12	75		6	37.5		8	50	
Anaemia	No	285	70.2	**0.039**	148	36.5	0.567	178	43.8	0.095
	Yes	257	76.9		115	34.4		167	50	
Asthma	No	477	72.5	0.191	235	35.7	0.780	299	45.4	0.068
	Yes	65	79.3		28	34.1		46	56.1	
Cardiac arrhythmia	No	218	68.8	**0.017**	107	33.8	0.379	137	43.2	0.108
	Yes	324	76.6		156	36.9		208	49.2	
Cerebrovascular disease (including hemiplegia)	No	397	71.9	0.164	200	36.2	0.501	252	45.7	0.365
	Yes	145	77.1		63	33.5		93	49.5	
Chronic obstructive pulmonary disease	No	334	71.5	0.166	168	36	0.747	213	45.6	0.471
	Yes	208	76.2		95	34.8		132	48.4	
Chronic gastritis or gastro-oesophageal reflux	No	472	73.1	0.774	219	33.9	**0.015**	302	46.7	0.855
	Yes	70	74.5		44	46.8		43	45.7	
Chronic renal insufficiency	No	303	72.1	0.439	143	34	0.331	190	45.2	0.387
	Yes	239	74.7		120	37.5		155	48.4	
Chronic thyroid disease	No	435	71.9	0.081	210	34.7	0.318	271	44.8	**0.035**
	Yes	107	79.3		53	39.3		74	54.8	
Degenerative arthropathy	No	244	68.7	**0.008**	105	29.6	**0.001**	149	42	**0.015**
	Yes	298	77.4		158	41		196	50.9	
Dementia	No	416	74.2	0.322	196	34.9	0.544	260	46.3	0.790
	Yes	126	70.4		67	37.4		85	47.5	
Diabetes with complication	No	442	72.8	0.576	221	36.4	0.292	286	47.1	0.564
	Yes	100	75.2		42	31.6		59	44.4	
Diabetes without complication	No	394	73.1	0.884	186	34.5	0.337	251	46.6	0.962
	Yes	148	73.6		77	38.3		94	46.8	
Drug-related conditions	No	491	73	0.577	241	35.8	0.628	314	46.7	0.952
	Yes	51	76.1		22	32.8		31	46.3	
Dyslipidaemia	No	268	70.5	0.086	143	37.6	0.222	175	46.1	0.75
	Yes	274	76.1		120	33.3		170	47.2	
Essential tremor	No	534	73.1	0.286	258	35.3	0.207	337	46.1	**0.011**
	Yes	8	88.9		5	55.6		8	88.9	
Fibromyalgia	No	536	73.2	0.91	260	35.5	0.907	340	46.4	0.365
	Yes	6	75		3	37.5		5	62.5	
Gallstones (previous hepatic colic)	No	482	72.9	0.565	226	34.2	**0.026**	312	47.2	0.361
	Yes	60	75.9		37	46.8		33	41.8	
Gout	No	443	73.5	0.774	213	35.3	0.796	287	47.6	0.265
	Yes	99	72.3		50	36.5		58	42.3	
Haematologic disorders	No	517	73.4	0.598	246	34.9	0.133	328	46.6	0.941
	Yes	25	69.4		17	47.2		17	47.2	
Heart failure	No	208	70	0.106	101	34	0.475	140	47.1	0.818
	Yes	334	75.4		162	36.6		205	46.3	
Hypertension	No	90	65.7	**0.027**	42	30.7	0.186	64	46.7	0.981
	Yes	452	75		221	36.7		281	46.6	
Inflammatory osteoarticular disease	No	509	73.7	0.335	237	34.3	**0.008**	324	46.9	0.585
	Yes	33	67.3		26	53.1		21	42.9	
Irritable bowel syndrome	No	531	72.8	**0.043**	258	35.4	0.489	338	46.4	0.254
	Yes	11	100		5	45.5		7	63.6	
Ischaemic heart disease without infarction	No	451	72.7	0.484	223	36	0.581	278	44.8	**0.027**
	Yes	91	75.8		40	33.3		67	55.8	
Migraine	No	541	73.5	**0.029**	262	35.6	0.659	344	46.7	0.385
	Yes	1	25		1	25		1	25	
Mild liver disease (incl. chronic hepatitis B or C)	No	516	72.9	0.296	248	35	0.171	327	46.2	0.264
	Yes	26	81.2		15	46.9		18	56.2	
Moderate or severe liver disease	No	534	74.1	**0.002**	259	35.9	0.181	342	47.4	**0.006**
	Yes	8	42.1		4	21.1		3	15.8	
Myocardial infarction	No	459	73	0.693	231	36.7	0.109	287	45.6	0.197
	Yes	83	74.8		32	28.8		58	52.3	
Neoplasia	No	469	74.6	0.054	224	35.6	0.923	295	46.9	0.718
	Yes	73	65.8		39	35.1		50	45	
Neurologic disorder of the central nervous system	No	521	73.6	0.32	254	35.9	0.37	328	46.3	0.451
	Yes	21	65.6		9	28.1		17	53.1	
Non-ischaemic heart disease	No	366	72.9	0.765	182	36.3	0.555	228	45.4	0.341
	Yes	176	73.9		81	34		117	49.2	
Non-schizophrenic mental disorders	No	532	73.1	0.426	257	35.3	0.291	341	46.8	0.352
	Yes	10	83.3		6	50		4	33.3	
Obesity	No	387	70.7	**0.01**	192	35.1	0.674	253	46.3	0.735
	Yes	155	80.3		71	36.8		92	47.7	
Osteoporosis	No	458	71.9	**0.04**	219	34.4	0.101	289	45.4	0.089
	Yes	84	81.6		44	42.7		56	54.4	
Pancreas disease	No	532	72.9	0.054	259	35.5	0.767	338	46.3	0.136
	Yes	10	100		4	40		7	70	
Parkinson's disease	No	517	73.2	0.969	252	35.7	0.691	327	46.3	0.45
	Yes	25	73.5		11	32.4		18	52.9	
Peptic ulcer disease	No	509	73.3	0.812	245	35.3	0.599	325	46.8	0.659
	Yes	33	71.7		18	39.1		20	43.5	
Peripheral neuropathy or neuritis	No	494	72.8	0.316	243	35.8	0.639	312	45.9	0.222
	Yes	48	78.7		20	32.8		33	54.1	
Peripheral vascular disease	No	461	72.6	0.330	232	36.5	0.164	295	46.5	0.825
	Yes	81	77.1		31	29.5		50	47.6	
Post-traumatic stress disorder	No	540	73.3	0.797	261	35.4	0.259	344	46.7	0.644
	Yes	2	66.7		2	66.7		1	33.3	
Previous fractures (not hip)	No	430	71.5	**0.03**	207	34.4	0.194	268	44.6	**0.021**
	Yes	112	80.6		56	40.3		77	55.4	
Previous hip fracture	No	488	72.5	0.154	231	34.3	**0.028**	312	46.4	0.651
	Yes	54	80.6		32	47.8		33	49.3	
Rheumatologic disease	No	522	73.7	0.16	255	36	0.203	332	46.9	0.487
	Yes	20	62.5		8	25		13	40.6	
Schizophrenia	No	540	73.3	0.797	262	35.5	0.936	343	46.5	0.486
	Yes	2	66.7		1	33.3		2	66.7	
Sleep apnoea	No	486	72.1	**0.026**	244	36.2	0.230	305	45.3	**0.017**
	Yes	56	84.8		19	28.8		40	60.6	
Tuberculosis	No	536	73.3	0.654	260	35.6	0.889	340	46.5	0.589
	Yes	6	66.7		3	33.3		5	55.6	
Urinary tract stones	No	530	73	0.287	259	35.7	0.582	340	46.8	0.409
	Yes	12	85.7		4	28.6		5	35.7	
Varicose veins	No	421	73.1	0.86	199	34.5	0.291	261	45.3	0.181
	Yes	121	73.8		64	39		84	51.2	
Vertigo	No	483	72.9	0.479	237	35.7	0.731	302	45.6	0.087
	Yes	59	76.6		26	33.8		43	55.8	

p<0.05 was considered statistically significant and highlighted in bold. PIP: potentially inappropriate prescribing. PIM: potentially inappropriate medication. PPO: potential prescribing omission. PPI: proton pump inhibitor. BZD: benzodiazepine.

*Benzodiazepines (BZDs) for ≥4 weeks* (STOPP criterion D5) was the second most frequent PIM, found in 31.8% of the patients. And the presence of any PIMs related to BZDs (STOPP criteria D5, G5, K1 or A1 involving BZDs) was found in 32.3%, with a high redundancy between these criteria.

Therefore, the most frequent active principles as PIMs were PPIs and BZDs, with 345 (46.6%) patients having at least one related PIM.

Regarding PPOs, at least one was identified in 263 (35.5%, 95% CI 32.2-39.1) patients, ranging from 0 to 4, with a median number of 0 (IQR 0-1). In total, 188 (25.4%) patients had 1 PPO, 62 (8.4%) had 2, 11 (1.5%) had 3, and 2 (0.3%) had 4 PPOs. The most frequent PPOs relative to the total of patients are summarized in Figure 1B, starting with *vitamin D supplement in older people who are housebound or experiencing falls or with osteopenia* (START criterion E5, 10.3%), followed by *laxatives in patients receiving opioids regularly* (H2, 6.8%), *beta-blockers with stable systolic heart failure* (A8, 5.3%) and *ACE inhibitors with systolic heart failure and/or documented coronary artery disease* (A6, 5.1%). All PPOs detected are shown in Supp. Table 3, relative to the total number of PPOs.

**Table 3:** Multilevel logistic regression model on the outcome of the presence of any most frequent active principles as PIMs (PPI or BZD)

Variable	Any most frequent active principles as PIMs (PPI/BZD) OR (95% CI)
Age 65-74	Reference
Age 75-89	1.75 (1.01, 3.09)
Age 90+	1.96 (1.05, 3.73)
Oligopharmacy (0-4)	Reference
Moderate polypharmacy (5-9)	3.03 (1.42, 7.01)
Excessive polypharmacy (10+)	5.12 (2.43, 11.77)
Essential tremor	19.21 (3.11, 374.95)
Previous fractures (not hip)	1.43 (0.94, 2.16)

PIM: potentially inappropriate medication. OR: odds ratio. CI: confidence interval. PPI: proton pump inhibitor. BZD: benzodiazepine.

### Factors associated to PIP

Next, we performed a bivariate analysis to uncover the potential relationship of sociodemographic and clinical variables with the prevalence of any PIM, any PPO and any most frequent active principles as PIMs (any PPI/BZD) (Tables 1 and 2).

All the significant variables obtained in the bivariate analysis of the outcome of any PPI/BZD as PIMs were included in a stepwise selection algorithm in order to build a multilevel logistic regression model. This explanatory model (Table 3) obtained a 71% AUC (95% CI 67.4-74.7) and showed contribution of age, polypharmacy, essential tremor and previous fractures excluding hip (not significant but necessary for optimal model). Remarkably, excessively polymedicated patients (>10 drugs) and those suffering from essential tremor were at least twice or trice more likely to have any PPI/BZD as PIMs, respectively (95% CI odds ratio lower limits >2 and >3).

## Discussion

Our study found a high proportion of older patients with an elevated rate of multimorbidity and moderate functional impairment, a high prevalence of polypharmacy (93.8%) (much higher than reported for the general Spanish population [26]), and a very high prevalence of excessive polypharmacy (58.8%). These findings are consistent with the inclusion of older patients admitted to hospital due to chronic disease exacerbation.

Regarding PIP, up to 81.5% of the patients met at least one criterion, mainly due to a high prevalence of PIMs (73.2%) instead of PPOs (35.5%). The prevalence of PIMs differs from the estimates of a recent systematic review in which 42.8% of the patients in the community presented at least a PIM, whereas the prevalence of PPOs is very similar [27]. It is plausible that patients in our cohort present a higher prevalence of PIMs due to their polypharmacy, multimorbidity, functional impairment and uncontrolled chronic problems. Besides, another factor could be the application of the STOPP/START criteria version 2, owing to STOPP criteria A (implicit), which may increase PIM detection but could be a possible source of variability too.

An important finding of this study is that the most frequent active principles as PIMs, which were PPIs and BZDs, were present in almost half (46.6%) of the patients, suggesting that actions focused on deprescribing these medications may have a large impact on reducing PIP and, therefore, undesired negative outcomes. Remarkably, many other studies have previously found either BZDs alone [28–30] or together with PPIs [4, 12, 31–33] among the most frequent PIMs.

With respect to PPIs, which are widely prescribed in Spain [34], they were classified as PIMs in 167 patients. PPIs may be related to adverse outcomes, such as fractures [35], hypomagnesaemia [36–39], recurrent *C. difficile* infection [40, 41], dementia [42, 43], community-acquired pneumonia [44], or severe COVID-19 infection [45–47]. Remarkably, in 160 (95.8%) patients, PPI prescription was assigned to implicit STOPP criterion A1. This situation may explain why other studies did not find a similar prevalence of PPIs as PIMs, since the pharmacists’ judgement becomes more relevant in implicit criteria.

The rest of active principles belonging to STOPP criterion A1 (which was indeed the most frequent PIM) were highly diverse, highlighting the need of more explicit criteria to avoid subjectivity in the screening, maybe at the expense of supressing criteria about less frequent situations, not to end up with an excessively long list.

Regarding BZDs, they are highly prescribed among older adults in Spain and their use has been increasing lately [48, 49]; however, its prescribing has been found significantly in excess of what the evidence would suggest is appropriate [50]. In fact, BZDs are associated with negative outcomes such as dependence, falls and fractures, cognitive decline or sleep disturbances [51].

Among the registered PPOs, vitamin D in older people who are experiencing falls or osteopenia was not expected to be the most frequent, but this could be partially explained by the strong levels of sun radiation in Spain. Furthermore, we encountered a high rate of patients not taking laxatives when consuming opioids, which could suppose a risk for constipation. The over-the-counter use of these drugs and/or herbal products (due to lack of prize reimbursement in Spain) may be a potential reason for this.

The bivariate analyses showed a significant association of the defined PIP outcomes with some sociodemographic and clinical variables such as age, polypharmacy and number of chronic conditions, which have been previously associated with the presence of PIM and PPO [31, 33, 52, 53]. Regarding specific chronic conditions, a large number showed an association, such as anaemia, degenerative arthropathy, sleep apnoea, inflammatory osteoarticular disease and previous hip fracture, among many others.

Finally, when modelling the presence of any PPI/BZD as PIMs, we found out the important role of age and polypharmacy, as expected, but also of two chronic conditions: essential tremor and previous fractures (excluding hip). Although these are not highly prevalent conditions, they have a role in the outcome. in fact, there is increasing evidence of a relationship between PPIs and fractures [35], which, together with the association of BZDs to falls and fractures [51], urges to review both PPIs and BZDs prescribing in these patients. Furthermore, the use of BZDs to treat essential tremor has shown a limited effectiveness [54].

Remarkably, the use of a multilevel logistic regression analysis provides more reliable results compared to conventional regression analyses. The latter consider that records of individual patients are independent of records of other patients. However, this assumption may not hold true in multicentre studies; for instance, different geographical areas may have variability in prescribing tendencies and patient profiles. Therefore, multilevel analyses, which allow to analyse data with a hierarchical structure, are appropriate to take these potential effects into account.

Previous, similar studies have been conducted aiming to find associations between chronic conditions and PIP outcomes. However, most have considered only a few comorbidities or risk factors, such as hypertension, dyslipidaemia, osteoporosis, diabetes or COPD [55, 56] and not a large, comprehensive list. Our findings highlight the need of a wider consideration of chronic conditions to incorporate to regression models, in order to detect subtler yet important associations. Regression models including chronic conditions can be useful to stratify patients according to their associated risk of presenting PIPs and, consequently, to identify which patients require a medication review priority.

### Clinical implications

Our results show how older patients admitted to hospital because of chronic conditions exacerbation present a higher prevalence of PIM compared to other cohorts from the community. Even though this study was carried out in a hospital setting, the medication review was performed the day of admission and, consequently, these were previous prescriptions originated from any facility in the whole healthcare system.

Patients with a larger number of chronic conditions have a higher probability of presenting any PIM or any of the most frequent active principles as PIMs (PPI/BZD). With these results, medication review could be more focused on these specific situations and drugs, given that it may not always be possible to conduct a medication review in all patients.

Interestingly, Barthel Index was also associated to PIP outcomes, but not in an increasing or decreasing tendency. In all three analysed outcomes (any PIM, any PPO, any PPI/BZD), independent patients or totally dependent ones (100 or <20 Barthel Index) presented the lowest prevalence of inappropriate prescription, whereas the group with highest prevalence of inappropriate prescription was that of severely dependent patients (20-35 Barthel Index). It is therefore possible that the patients at the “extremes” have less PIP because there are more actions directed to medication review in these cases.

These results highlight the need of a thorough medication review in which the hospital pharmacists are integrated within the multidisciplinary geriatric team. With this approach, clinical practice quality could be improved.

### Strengths and limitations

The strengths of this study are its multicentre, prospective design in a hospital setting covering different regions of Spain, a team of trained pharmacists integrated in multidisciplinary teams with geriatricians or internal medicine practitioners [57] already familiar with the STOPP/START screening tool, as well as the assurance of high quality and thoroughness in all the gathered clinical and pharmacological data. The study sample size has enough power to estimate the prevalence of PIP, PIM and PPO and is proportional to the volume of admissions of each hospital. Furthermore, the use of a large, comprehensive list of chronic conditions as possible factors associated with PIP as well as an outcome variable that focuses on the presence of the most common misprescriptions are the most powerful strengths of this work.

However, this study also presents some limitations. The application of STOPP/START criteria by different centres and professionals may have induced some biases, especially in those implicit criteria. For this reason, each participating hospital was set as a first level in the multilevel logistic regression model. Moreover, the lack of data on vaccines may affect the prevalence of PPOs. Nonetheless, vaccination is entirely different than the rest of PPOs and therefore the outcome variable excluding vaccines is still clinically and pharmacologically coherent.

## Conclusions

The findings of the study confirm that there is a high prevalence of PIP at admission in older, hospitalized patients due to chronic disease exacerbation mainly by the inappropriate prescription of PPIs or BZDs. These drugs have been associated to a set of different chronic conditions as well as age and polypharmacy, giving a starting point for medication review and deprescription. Thus, our study identified a patient profile with higher risk of PIP towards which these actions should be focused. Finally, our results highlight the essentiality of multidisciplinary teams in the clinical management of these patients.

## Supplementary Information

**Additional file 1.**

## Data Availability

The datasets used and/or analysed during the current study are available from the corresponding author on reasonable request.

## Abbreviations

PIP: Potentially inappropriate prescribing
PIM: Potentially inappropriate medication
PPO: Potential prescribing omission
BZD: Benzodiazepine
PPI: Proton pump inhibitor
AUC: Area under the curve
IQR: interquartile range
CI: confidence interval
