# Supplementary material for: Factors associated to potentially inappropriate prescribing in older patients according to STOPP/START criteria: MoPIM multicentre cohort study
Source: BMC Geriatr. 2022 Jan 11;22:44. doi: 10.1186/s12877-021-02715-8 (PMC8751453; doi:10.1186/s12877-021-02715-8)
Supplement: Supplementary file 1 — Additional file 1. [file 12877_2021_2715_MOESM1_ESM.docx]

| Active principle / Drug class | N | % |
| --- | --- | --- |
| **Proton pump inhibitor** | **160** | **51.6** |
| Esomeprazole | 3 | 1 |
| Lansoprazole | 2 | 0.6 |
| Omeprazole | 144 | 46.5 |
| Pantoprazole | 10 | 3.2 |
| Rabeprazole | 1 | 0.3 |
| **Hypolipidemic drug** | **29** | **9.4** |
| Atorvastatin | 8 | 2.6 |
| Lovastatin | 1 | 0.3 |
| Simvastatin | 20 | 6.5 |
| **Analgesic drug** | **24** | **7.7** |
| Codeine | 1 | 0.3 |
| Metamizole | 2 | 0.6 |
| Paracetamol | 18 | 5.8 |
| Tramadol | 3 | 1 |
| **Aspirin** | **16** | **5.2** |
| **Antihypertensive drug** | **10** | **3.2** |
| Delapril | 1 | 0.3 |
| Enalapril | 2 | 0.6 |
| Furosemide | 2 | 0.6 |
| Losartan | 3 | 1 |
| Telmisartan | 1 | 0.3 |
| Valsartan | 1 | 0.3 |
| **Other** | **71** | **22.9** |
| Acetylcisteine | 1 | 0.3 |
| Allopurinol | 9 | 2.9 |
| Amitriptyline | 1 | 0.3 |
| Betahistine | 1 | 0.3 |
| Calcium | 1 | 0.3 |
| Calcium and Vitamin D | 1 | 0.3 |
| Calcium folinate | 1 | 0.3 |
| Carvedilol | 1 | 0.3 |
| Clopidogrel | 3 | 1 |
| Cyanocobalamin | 1 | 0.3 |
| Chondroitin sulphate | 1 | 0.3 |
| Desloratadine | 1 | 0.3 |
| Diclofenac | 1 | 0.3 |
|  |  |  |
| Doxylamine | 1 | 0.3 |
| Dutasteride | 1 | 0.3 |
| Flecainide | 1 | 0.3 |
| Folic Acid | 2 | 0.6 |
| Gabapentin | 1 | 0.3 |
| Glucosamine | 1 | 0.3 |
| Hydroxyzine | 1 | 0.3 |
| Iron | 1 | 0.3 |
| Ivabradine | 2 | 0.6 |
| Levodopa/carbidopa | 1 | 0.3 |
| Lidocaine | 1 | 0.3 |
| Loratadine | 1 | 0.3 |
| Lorazepam | 2 | 0.6 |
| Memantine | 1 | 0.3 |
| Metformin | 1 | 0.3 |
| Mirtazapine | 1 | 0.3 |
| Naphthydrofuryl | 1 | 0.3 |
| Nitroglycerin | 1 | 0.3 |
| Paroxetine | 1 | 0.3 |
| Pentoxifylline | 3 | 1 |
| Potassium | 2 | 0.6 |
| Potassium aspartate | 1 | 0.3 |
| Potassium bicarbonate | 1 | 0.3 |
| Prednisone | 1 | 0.3 |
| Propafenone | 1 | 0.3 |
| Quetiapine | 1 | 0.3 |
| Ranolazine | 3 | 1 |
| Sabal serrulata | 1 | 0.3 |
| Salbutamol | 1 | 0.3 |
| Serenoa repens | 1 | 0.3 |
| Sertraline | 4 | 1.3 |
| Solifenacin | 1 | 0.3 |
| Tamsulosin | 1 | 0.3 |
| Trazodone | 2 | 0.6 |
| Trimetazidine | 1 | 0.3 |
| Vitamin B12 | 1 | 0.3 |
| **Total** | **310** | **100** |

Supp.Table 1. Number and percentage of the STOPP A1 criterion active principles and the created subcategories (proton pump inhibitors, hypolipidemic drugs, analgesic drugs aspirin, antihypertensive drugs).

| Code_description | N | % |
| --- | --- | --- |
| A1: Any drug prescribed without an evidence-based clinical indication | 310 | 25.70 |
| A2: Any drug prescribed beyond the recommended duration, where treatment duration is well defined | 40 | 3.32 |
| A3: Any duplicate drug class prescription | 31 | 2.57 |
| B1: Digoxin for heart failure with normal systolic ventricular function | 5 | 0.41 |
| B2: Verapamil or diltiazem with NYHA Class III or IV heart failure | 5 | 0.41 |
| B3: Beta-blocker in combination with verapamil or diltiazem | 1 | 0.08 |
| B4: Beta blocker with bradycardia, type II heart block or complete heart block | 3 | 0.25 |
| B5: Amiodarone as first-line antiarrhythmic therapy in supraventricular tachyarrhythmia | 13 | 1.08 |
| B6: Loop diuretic as first-line treatment for hypertension | 13 | 1.08 |
| B7: Loop diuretic for dependent ankle oedema without clinical, biochemical evidence or radiological evidence of heart failure, liver failure, nephrotic syndrome or renal failure | 8 | 0.66 |
| B8: Thiazide diuretic with current significant hypokalaemia, hyponatraemia, hypercalcaemia or with a history of gout | 18 | 1.49 |
| B9: Loop diuretic for treatment of hypertension with concurrent urinary incontinence | 5 | 0.41 |
| B11: ACE inhibitors or Angiotensin Receptor Blockers in patients with hyperkalaemia | 45 | 3.73 |
| B12: Aldosterone antagonists with concurrent potassium-conserving drugs without monitoring of serum potassium | 18 | 1.49 |
| C1: Long-term aspirin at doses greater than 160mg per day | 14 | 1.16 |
| C2: Aspirin with a past history of peptic ulcer disease without concomitant PPI | 4 | 0.33 |
| C3: Aspirin, clopidogrel, dipyridamole, vitamin K antagonists, direct thrombin inhibitors or factor Xa inhibitors with concurrent significant bleeding risk | 5 | 0.41 |
| C4: Aspirin plus clopidogrel as secondary stroke prevention, unless the patient has a coronary stent(s) inserted in the previous 12 months or concurrent acute coronary syndrome or has a high grade symptomatic carotid arterial stenosis | 3 | 0.25 |
| C5: Aspirin in combination with vitamin K antagonist, direct thrombin inhibitor or factor Xa inhibitors in patients with chronic atrial fibrillation | 8 | 0.66 |
| C6: Antiplatelet agents with vitamin K antagonist, direct thrombin inhibitor or factor Xa inhibitors in patients with stable coronary, cerebrovascular or peripheral arterial disease | 2 | 0.17 |
| C8: Vitamin K antagonist, direct thrombin inhibitor or factor Xa inhibitors for first deep venous thrombosis without continuing provoking risk factors for > 6 months | 2 | 0.17 |
| C9: Vitamin K antagonist, direct thrombin inhibitor or factor Xa inhibitors for first pulmonary embolus without continuing provoking risk factors for > 12 months | 3 | 0.25 |
| C10: NSAID and vitamin K antagonist, direct thrombin inhibitor or factor Xa inhibitors in combination | 5 | 0.41 |
| D1: TriCyclic Antidepressants (TCAs) with dementia, narrow angle glaucoma, cardiac conduction abnormalities, prostatism, or prior history of urinary retention | 7 | 0.58 |
| D2: Initiation of TriCyclic Antidepressants (TCAs) as first-line antidepressant treatment | 4 | 0.33 |
| D3: Neuroleptics with moderate-marked antimuscarinic/anticholinergic effects with a history of prostatism or previous urinary retention | 2 | 0.17 |
| D4: Selective serotonin re-uptake inhibitors (SSRI's) with current or recent significant hyponatraemia i.e. serum Na+ < 130 mmol/l | 5 | 0.41 |
| D5: Benzodiazepines for ≥ 4 weeks | 247 | 20.48 |
| D7: Anticholinergics/antimuscarinics to treat extra-pyramidal side-effects of neuroleptic medications | 1 | 0.08 |
| D8: Anticholinergics/antimuscarinics in patients with delirium or dementia | 8 | 0.66 |
| D9: Neuroleptic antipsychotic in patients with behavioural and psychological symptoms of dementia (BPSD) unless symptoms are severe and other non-pharmacological treatments have failed | 3 | 0.25 |
| D10: Neuroleptics as hypnotics, unless sleep disorder is due to psychosis or dementia | 2 | 0.17 |
| D11: Acetylcholinesterase inhibitors with a known history of persistent bradycardia, heart block or recurrent unexplained syncope or concurrent treatment with drugs that reduce heart rate | 1 | 0.08 |
| D14: First-generation antihistamines | 3 | 0.25 |
| E1: Digoxin at a long-term dose greater than 125Âµg/day if eGFR < 30 ml/min/1.73m2 | 3 | 0.25 |
| E2: Direct thrombin inhibitors (e.g. dabigatran) if eGFR < 30 ml/min/1.73m2 | 0 | 0.00 |
| E3: Factor Xa inhibitors (e.g. rivaroxaban, apixaban) if eGFR < 15 ml/min/1.73m2 | 0 | 0.00 |
| E4: NSAID's if eGFR < 50 ml/min/1.73m2 | 9 | 0.75 |
| E5: Colchicine if eGFR < 10 ml/min/1.73m2 | 0 | 0.00 |
| E6: Metformin if eGFR < 30 ml/min/1.73m2 | 9 | 0.75 |
| F1: Prochlorperazine or metoclopramide with Parkinsonism | 0 | 0.00 |
| F2: PPI for uncomplicated peptic ulcer disease or erosive peptic oesophagitis at full therapeutic dosage for > 8 weeks | 7 | 0.58 |
| F3: Drugs likely to cause constipation in patients with chronic constipation where non-constipating alternatives are available | 1 | 0.08 |
| F4: Oral elemental iron doses greater than 200 mg daily | 2 | 0.17 |
| G1: Theophylline as monotherapy for COPD | 0 | 0.00 |
| G2: Systemic corticosteroids instead of inhaled corticosteroids for maintenance therapy in moderate-severe COPD | 2 | 0.17 |
| G3: Anti-muscarinic bronchodilators (e.g. ipratropium, tiotropium) with a history of narrow angle glaucoma or bladder outflow obstruction | 2 | 0.17 |
| G4: Non-selective beta-blocker (whether oral or topical for glaucoma) with a history of asthma requiring treatment | 4 | 0.33 |
| G5: Benzodiazepines with acute or chronic respiratory failure i.e. pO2 < 8.0 kPa Â± pCO2 > 6.5 kPa | 54 | 4.48 |
| H1: Non-steroidal anti-inflammatory drug (NSAID) other than COX-2 selective agents with history of peptic ulcer disease or gastrointestinal bleeding, unless with concurrent PPI or H2 antagonist | 0 | 0.00 |
| H2: NSAID with severe hypertension or severe heart failure | 5 | 0.41 |
| H3: Long-term use of NSAID (>3 months) for symptom relief of osteoarthritis pain where paracetamol has not been tried | 1 | 0.08 |
| H5: Corticosteroids (other than periodic intra-articular injections for mono-articular pain) for osteoarthritis | 1 | 0.08 |
| H7: COX-2 selective NSAIDs with concurrent cardiovascular disease | 1 | 0.08 |
| I1: Antimuscarinic drugs with dementia, or chronic cognitive impairment or narrow-angle glaucoma, or chronic prostatism | 12 | 1.00 |
| I2: Selective alpha-1 selective alpha blockers in those with symptomatic orthostatic hypotension or micturition syncope | 1 | 0.08 |
| J1: Sulphonylureas with a long duration of action (e.g. glibenclamide, chlorpropamide, glimepiride) with type 2 diabetes mellitus | 4 | 0.33 |
| J3: Beta-blockers in diabetes mellitus with frequent hypoglycaemic episodes | 3 | 0.25 |
| K1: Benzodiazepines | 131 | 10.86 |
| K2: Neuroleptic drugs | 32 | 2.65 |
| K3: Vasodilator drugs with persistent postural hypotension i.e. recurrent drop in systolic blood pressure ≥ 20mmHg | 6 | 0.50 |
| K4: Hypnotic Z-drugs e.g. zopiclone, zolpidem, zaleplon | 10 | 0.83 |
| L1: Use of oral or transdermal strong opioids as first line therapy for mild pain | 17 | 1.41 |
| L2: Use of regular (as distinct from PRN) opioids without concomitant laxative | 40 | 3.32 |
| L3: Long-acting opioids without short-acting opioids for break-through pain | 3 | 0.25 |
| N1: Concomitant use of two or more drugs with antimuscarinic/anticholinergic properties | 2 | 0.17 |
| Total | 1206 | 100.00 |

Supp.Table 2. Number and percentage of PIMs according to STOPP criteria.

| Code_description | N | % |
| --- | --- | --- |
| A1: Vitamin K antagonists or direct thrombin inhibitors or factor Xa inhibitors in the presence of chronic atrial fibrillation | 20 | 5.67 |
| A2: Aspirin (75 mg – 160 mg once daily) in the presence of chronic atrial fibrillation, where Vitamin K antagonists or direct thrombin inhibitors or factor Xa inhibitors are contraindicated | 2 | 0.57 |
| A3: Antiplatelet therapy (aspirin or clopidogrel or prasugrel or ticagrelor) with a documented history of coronary, cerebral or peripheral vascular disease | 9 | 2.55 |
| A4: Antihypertensive therapy where systolic blood pressure consistently > 160 mmHg and/or diastolic blood pressure consistently >90 mmHg; if systolic blood pressure > 140 mmHg and /or diastolic blood pressure > 90 mmHg, if diabetic | 9 | 2.55 |
| A5: Statin therapy with a documented history of coronary, cerebral or peripheral vascular disease, unless the patient's status is end-of-life or age is > 85 years | 3 | 0.85 |
| A6: Angiotensin Converting Enzyme (ACE) inhibitor with systolic heart failure and/or documented coronary artery disease | 38 | 10.76 |
| A7: Beta-blocker with ischaemic heart disease | 14 | 3.97 |
| A8: Appropriate beta-blocker (bisoprolol, nebivolol, metoprolol or carvedilol) with stable systolic heart failure | 39 | 11.05 |
| B1: Regular inhaled b2 agonist or antimuscarinic bronchodilator (e.g. ipratropium, tiotropium) for mild to moderate asthma or COPD | 2 | 0.57 |
| B2: Regular inhaled corticosteroid for moderate-severe asthma or COPD, where FEV1 <50% of predicted value and repeated exacerbations requiring treatment with oral corticosteroids | 3 | 0.85 |
| B3: Home continuous oxygen with documented chronic hypoxaemia (i.e. pO2 < 8.0 kPa or 60 mmHg or SaO2 < 89%) | 0 | 0.00 |
| C1: L-DOPA or a dopamine agonist in idiopathic Parkinson's disease with functional impairment and resultant disability | 0 | 0.00 |
| C2: Non-TCA antidepressant drug in the presence of persistent major depressive symptoms | 14 | 3.97 |
| C3: Acetylcholinesterase inhibitor (e.g. donepezil, rivastigmine, galantamine) for mild-moderate Alzheimer's dementia or Lewy Body dementia (rivastigmine) | 9 | 2.55 |
| C4: Topical prostaglandin, prostamide or beta-blocker for primary open-angle glaucoma | 0 | 0.00 |
| C5: Selective serotonin reuptake inhibitor (or SNRI or pregabalin if SSRI contraindicated) for persistent severe anxiety that interferes with independent functioning | 2 | 0.57 |
| C6: Dopamine agonist (ropinirole or pramipexole or rotigotine) for Restless Legs Syndrome, once iron deficiency and severe renal failure have been excluded | 0 | 0.00 |
| D1: Proton Pump Inhibitor with severe gastro-oesophageal reflux disease or peptic stricture requiring dilatation | 6 | 1.70 |
| D2: Fibre supplements (e.g. bran, ispaghula, methylcellulose, sterculia) for diverticulosis with a history of constipation | 2 | 0.57 |
| E1: Disease-modifying anti-rheumatic drug (DMARD) with active, disabling rheumatoid disease | 1 | 0.28 |
| E2: Bisphosphonates and vitamin D and calcium in patients taking long-term systemic corticosteroid therapy | 10 | 2.83 |
| E3: Vitamin D and calcium supplement in patients with known osteoporosis and/or previous fragility fracture(s) and/or (Bone Mineral Density T-scores more than -2.5 in multiple sites) | 21 | 5.95 |
| E4: Bone anti-resorptive or anabolic therapy (e.g. bisphosphonate, strontium ranelate, teriparatide, denosumab) in patients with documented osteoporosis, where no pharmacological or clinical status contraindication exists (Bone Mineral Density T-scores -> 2.5 in multiple sites) and/or previous history of fragility fracture(s). | 7 | 1.98 |
| E5: Vitamin D supplement in older people who are housebound or experiencing falls or with osteopenia (Bone Mineral Density T-score is > -1.0 but < -2.5 in multiple sites) | 76 | 21.53 |
| E6: Xanthine-oxidase inhibitors (e.g. allopurinol, febuxostat) with a history of recurrent episodes of gout | 3 | 0.85 |
| E7: Folic acid supplement in patients taking methotexate | 1 | 0.28 |
| F1: ACE inhibitor or Angiotensin Receptor Blocker (if intolerant of ACE inhibitor) in diabetes with evidence of renal disease i.e. dipstick proteinuria or microalbuminuria (>30mg/24 hours) with or without serum biochemical renal impairment. | 4 | 1.13 |
| G1: Alpha-1 receptor blocker with symptomatic prostatism, where prostatectomy is not considered necessary | 2 | 0.57 |
| G2: 5-alpha reductase inhibitor with symptomatic prostatism, where prostatectomy is not considered necessary | 4 | 1.13 |
| G3: Topical vaginal oestrogen or vaginal oestrogen pessary for symptomatic atrophic vaginitis | 0 | 0.00 |
| H1: High-potency opioids in moderate-severe pain, where paracetamol, NSAIDs or low-potency opioids are not appropriate to the pain severity or have been ineffective | 2 | 0.57 |
| H2: Laxatives in patients receiving opioids regularly | 50 | 14.16 |
| Total | 353 | 100.00 |

Supp.Table 3. Number and percentage of PPOs according to START criteria.
